# Rapid Growth of Metal–Metal Oxide Core–Shell Structures through Joule Resistive Heating: Morphological, Structural, and Luminescence Characterization

**DOI:** 10.3390/ma17010208

**Published:** 2023-12-30

**Authors:** Juan Francisco Ramos-Justicia, Ana Urbieta, Paloma Fernández

**Affiliations:** Department of Materials Physics, Faculty of Physics, Complutense University of Madrid, 28040 Madrid, Spain; juanfrra@ucm.es (J.F.R.-J.); anaur@ucm.es (A.U.)

**Keywords:** resistive heating, metal oxides, core–shell composites, fast growth, defects

## Abstract

The aim of this study is to prove that resistive heating enables the synthesis of metal/metal oxide composites in the form of core–shell structures. The thickness and morphology of the oxide layer depends strongly on the nature of the metal, but the influences of parameters such as the time and current profiles and the presence of an external field have also been investigated. The systems chosen for the present study are Zn/ZnO, Ti/TiO_2_, and Ni/NiO. The characterization of the samples was performed using techniques based on scanning electron microscopy (SEM). The thicknesses of the oxide layers varied from 10 μm (Zn/ZnO) to 50 μm (Ni/NiO). In the case of Zn- and Ti-based composites, the growth of nanostructures on the oxide layer was observed. Micro- and nanoneedles formed on the ZnO layer while prism-like structures appeared on the TiO_2_. In the case of the NiO layer, micro- and nanocrystals were observed. Applying an external electric field seemed to align the ZnO needles, whereas its effect on TiO_2_ and NiO was less appreciable, principally affecting the shape of their grain boundaries. The chemical compositions were analysed using X-ray spectroscopy (EDX), which confirmed the existence of an oxide layer. Structural information was obtained by means of X-ray diffraction (XRD) and was later checked using Raman spectroscopy. The oxide layers seemed to be crystalline and, although some non-stoichiometric phases appeared, the stoichiometric phases were predominant; these were wurtzite, rutile, and cubic for Zn, Ti, and Ni oxides, respectively. The photoluminescence technique was used to study the distribution of defects on the shell, and mainly visible bands (2–2.5 eV), attributed to oxygen vacancies, were present. The near-band edges of ZnO and TiO_2_ were also observed around 3.2–3.3 eV.

## 1. Introduction

Transition metal oxides (TMO) are becoming more important for society every day because their properties grant them a huge versatility and applicability in a broad range of fields. Applications such as the degradation of water pollutants, environmental remediation, the photocatalytic reduction of CO_2_, the generation of organic light-emitting diodes (OLED) or efficient high-power conversion devices are some examples which illustrate the unavoidable urgency of improving these materials to advance towards a cleaner world, one which has been poisoned and endangered by humanity [1,2]. Nevertheless, a deeper comprehension of the physical phenomena is needed to transfer their properties to possible applications, since these properties depend strongly on morphology, doping, composition, intrinsic defects, etc. [2]. On the other hand, in most cases, to attain the optimal properties it is necessary to properly combine the virtues of each TMO. This has led to the study of composite materials, whose underlying idea lies in synergizing two different materials to improve the properties of the resultant hybrid with respect to the features of each of them separately. Although the possibilities for combining materials are almost infinite, the “active” part in all TMO-based composites is the oxide. The most frequent geometries of these composites are heterostructures, arrays of them, or core–shell structures [3,4]. In this work, we will focus on zinc, titanium, and nickel oxides because they share some features that make them appealing to face some of the challenges of the present.

Zinc oxide (ZnO) has been known by humanity since time immemorial; however, its uses have changed over time. Its most recent uses rely on its wide band gap (3.37 eV at ambient temperature) and its large exciton energy (60 meV). ZnO is used in absorbers of UV radiation introduced in clothes or tanning lotions, and even in solar cells [5,6,7]. It has also been proved that ZnO has good resistance towards microorganisms and antimicrobial properties, attributed to the generation of reactive oxygen species (ROS) [8]. Moreover, ZnO has potential applications in highly demanding industrial areas including the development of photo-catalysts for the degradation of pollutants. In the same vein, titanium oxide (TiO_2_) is the best photocatalyst discovered so far, and this excellent property has prompted thorough, extensive research. Other biomedical applications directly related to the photocatalysis include air purification, water removal, drug delivery, and biosensors [9]. Its usage as bleacher and filler material is remarkable as well [10]. Titanium oxide plays a crucial role both in photocatalysis and antimicrobial activity, but many questions still remain open [11]. Finally, NiO is also studied in this work. Although NiO is also a very promising material for most of the mentioned applications, it has not been so extensively investigated [12].

The present work deals with the growth of core–shell structures (Zn/ZnO, Ti/TiO_2_, and Ni/NiO) using the resistive heating method. This route has several advantages which traditional synthesis does not have, such as a low cost, short growth time, and ease of use. When an electrical current passes through a wire in ambient conditions, it heats up (in a process called the “Joule effect”), and if the temperature is high enough, it oxidizes and creates an oxide layer around it. This method has already been proved for ZrO_2_, ZnO, and CuO [4,13,14]; although the growth mechanism is still controversial, diffusion and electromigration are good candidates to control the oxidation process and the growth of nanostructures [14,15,16]. In this work, we will investigate the influence of different factors on the morphology of the layer, mainly the time-current profiles and the application of an external electric field. Exhaustive research concerning the optimization of control parameters (time, electric current, external electric field) and the study of morphology, crystalline structures, the chemical composition of the composite layer, and luminescence properties has been performed. If the three oxides selected are very adequate to create novel composites structures with improved catalytic properties (with photocatalysis and gas sensing applications), then we must first assess whether the individual oxides grown via fast Joule heating have the desired properties prior to the fabrication of more complex systems.

## 2. Experimental Method

**Figure 1:**
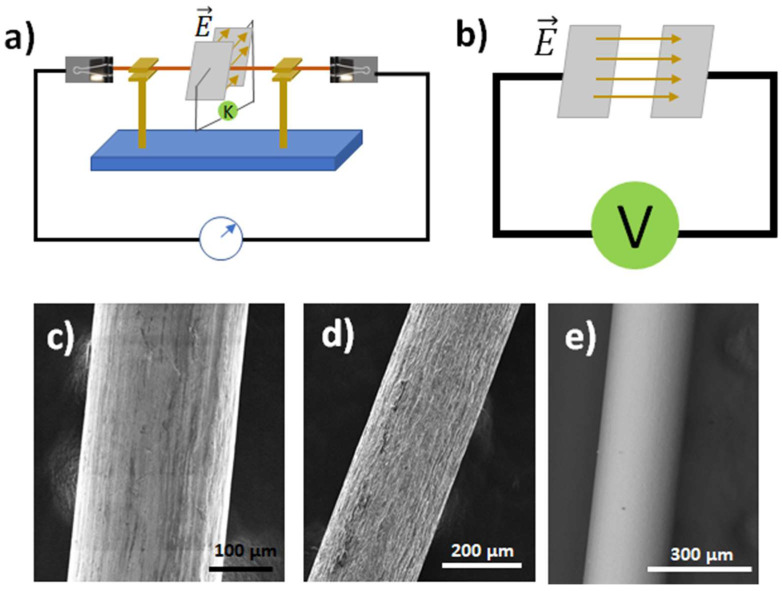
(**a**) Sketch of the experimental setup for growing samples. A wire (brown) is suspended between two electrodes connected to a source (circle with a blue arrow symbol). Optionally, two plates connected to the external source can activate an external electric field. The image design is adapted from [13]; (**b**) Sketch of the setup providing the electric field (brown arrows). In the second row, topographical images of (**c**) Zn wire, (**d**) Ti wire, and (**e**) Ni wire.

The experimental method that has been used to grow core–shell structures is illustrated in Figure 1a. A metallic wire is set between two electrodes connected to a power source. The electric current is driven through the wire, which causes a temperature gradient along it and promotes its oxidation via the Joule effect [13]. In this way, an oxide shell can be formed around the wire, yielding core–shell structures. The thickness of the layer depends on the nature of the wire. To study the effect of an external electric field [17], a detachable gadget is used, which consists of two movable metal plates, both perpendicular to the wire axis. When two metallic plates have different voltages, an electric field is generated between them, as shown in Figure 1b. This field is also uniform, and its modulus can be calculated as E = V/d, where V is the voltage between the plates and d is their distance. The voltage between plates can vary from 0 V to 210 V. In our case, we selected V = 210 V and d = 2 cm, meaning E = 10,500 V/m; we then studied the effects of the electric field on the part of the wire passing between the plates. This experimental set up has been used previously, as reported by Hidalgo et al. [14,18] and Zhao et al. [19].

The shown polycrystalline metallic wires are commercially available (zinc (ThermoScientific Chemicals (Waltham, MA, USA), X18F005), titanium (Goodfellow (Huntingdon, England) TI00-WR-000123), and nickel (Goodfellow NI00-WR-000140)) with purities of 99.99%, 99.6%, and 99%, respectively. Some SEM images of the pure wires (Figure 1c–e) have been taken. As will be shown later, there are no relevant impurities affecting our results. The wire length (measured from one electrode to the other) is 10 cm and the diameter is 0.25 mm in all cases.

**Figure 2:**
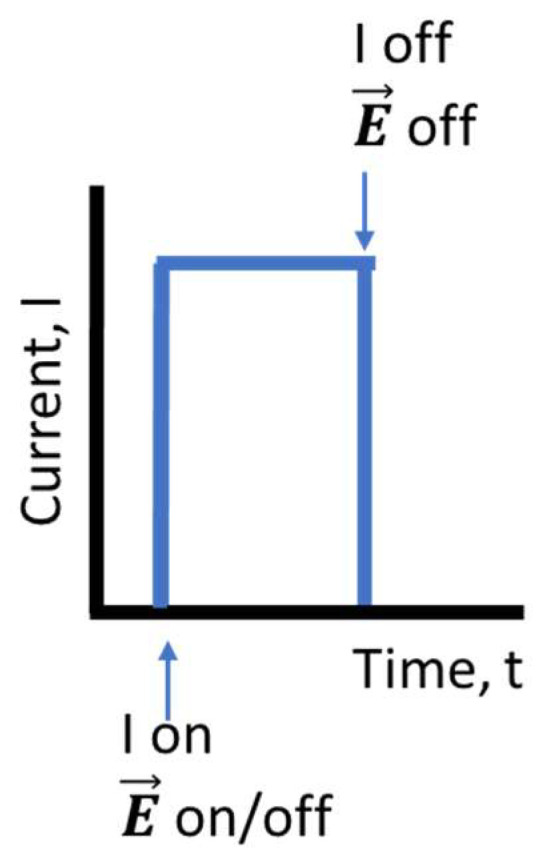
Sketch of the general current–time profiles used to grow samples.

The growth parameters influencing the oxide layer are mainly the current and time [17,20]; however, in this work, the effect of an external electric field during the formation of the oxide shell was also studied. Figure 2 depicts a basic sketch of the current–time profile. Firstly, the source supplying the electric current and, optionally, the electrical field generator are switched on. Secondly, the current intensity driven through the wire is kept constant at all times. Lastly, both the current and field, if present, sources are switched off. The first step is the determination of the current and time conditions that optimize the growth of the oxide layer. To study how they affect the formation, morphology, and structures of the oxide layer, different sets of samples were prepared. Table 1 summarizes the growth conditions of each wire. The nomenclature of samples has been chosen according to the chemical symbol of the element composing the wire. If an external electric field was applied, the name is indicated with an “E”. An electric field was applied only for the samples grown under the optimum current and time conditions.

The characterization of the samples was performed using SEM techniques and optical spectroscopy. Emissive mode images were taken using an FEI Inspect SEM (FEI Company, Hillsboro, OR, USA). X-ray microanalysis (EDX) was performed using a Hitachi TM 3000 SEM (Japan) equipped with a Brucker AXS Quantax system (United Kingdom). Microphotoluminescence (μ-PL) and µ-Raman spectra were obtained by means of a Horiba Jobin-Ybon LabRAM HR800 (Horiba Jobin Yvon LabRAM HR800, Villeneuve d’Ascq, France) with an excitation He–Cd laser source at 325 nm (PL and Raman) and an He–Ne laser source at 633 nm (Raman). The crystal structure was studied using X-ray diffraction using a PANalytical Empyrean with a Cu–K_α_ line. The electrical field was applied using a Keithley 2400 Sourcemeter (USA) as the power source, and the oxidation current was supplied using an EA-PS 3016-40 B source (Viersen, Germany).

## 3. Results and Discussion

**Figure 3:**
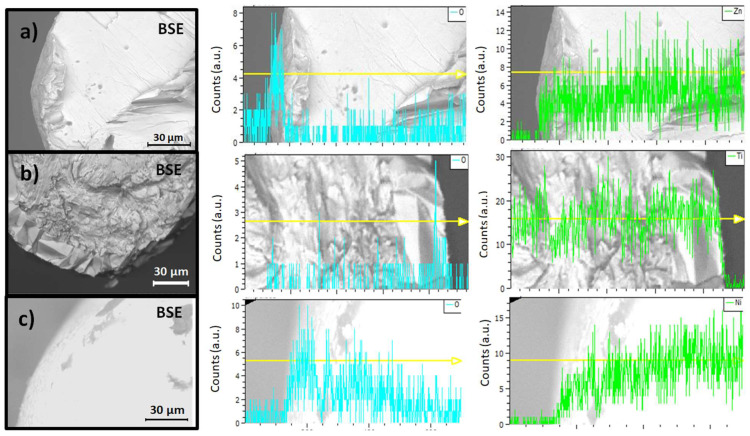
Sorted by rows, details of the cross sections of the (**a**) Zn family, (**b**) Ti family, and (**c**) Ni family. In all cases, the columns show: a topographical image (**left**), the oxygen profile (**center**), and the metal profile (**right**). The oxygen signal, larger at the outer surface, evidences the presence of an oxide layer at the boundaries of the samples.

**Figure 4:**
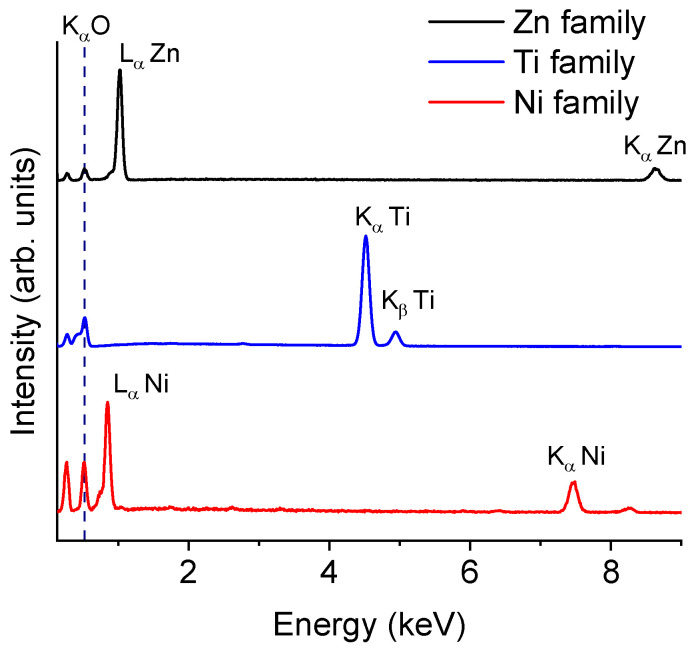
EDX spectra of the surface of the wires belonging to the (**up**) Zn family, (**center**) Ti family, and (**down**) Ni family. All spectra were recorded at 10 keV.

The morphology and composition of the core/shell structures were investigated using SEM (emissive mode and X-ray microanalysis, respectively). Figure 3 shows details of the cross sections of wires from the Zn, Ti, and Ni families and maps the homogeneous distribution of oxygen and the metal making up the core. As shown in the profiles, oxygen is only detected at the boundary of the wire, whose existence proves the presence of an external oxide layer. These mappings also enable us to calculate an estimation of the thickness of each wire by averaging some of the radii of the oxide circular crown; those belonging to the Zn family are approximately 10 μm thick, those in the Ti family are 30 μm thick, and the widths of the oxide layers of Ni samples range from 40 to 50 μm, approximately. X-ray microanalysis spectra of the wire shells (EDX) are shown in Figure 4. Despite the variety of growth conditions, there are no differences between them, so we only plotted one representative spectrum for each family. All spectra were recorded at 10 keV to minimize the impact of the metal core on the recorded signal. The peak situated at 0.52 keV, attributed to oxygen, is common to all spectra, proving the existence an oxide layer and, consequently, the correct formation of a core–shell composite. There are also other peaks common to all spectra, such as that at 0.27 keV, which corresponds to the K_α_ line of carbon (C). In part a of Figure 4, peaks at 1.01 keV and 8.63 keV belong to the L_α_ and K_α_ lines of zinc (Zn), respectively, which agrees with the bibliography [21]. Part b of the figure shows a typical spectrum of titanium oxide, as can be found in [22]. Peaks at 4.54 keV and 4.95 keV are attributed to the K_α_ and K_β_ lines of titanium (Ti). In part c, a peak at 0.84 keV is observed which is associated with the L_α_ line of nickel (Ni). A shoulder around 0.77 keV is also present and may be ascribed to the L–M shell transitions of nickel. The peak at 7.50 keV belongs to the K_α_ line of nickel. Finally, the small peak shown at 8.28 keV could be ascribed to a K_α_–M nickel shell transition. All these results agree with prior studies concerning NiO [23]. In all cases, there are no peaks attributed to wire impurities. The main difference between the spectra of the oxide layer and those of the core (not shown) lies in the presence of the 0.52 keV peak characteristic of the oxygen constituting metal oxides.

**Figure 5:**
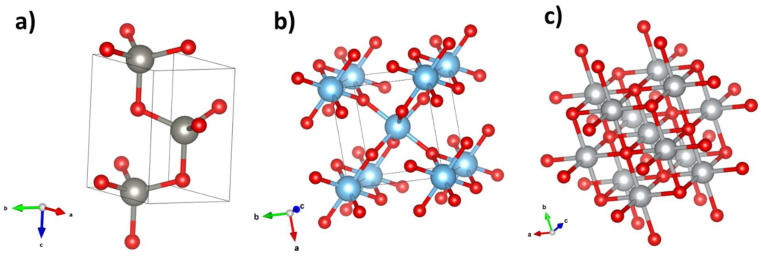
Crystal structure of (**a**) ZnO wurtzite (Zn in grey, O in red), (**b**) rutile (Ti in blue, O in red), (**c**) cubic NiO (Ni in grey, O in red).

The expected crystal structures of the three oxides studied, generated using VESTA 4.6.0 [24], are shown in Figure 5.

**Figure 6:**
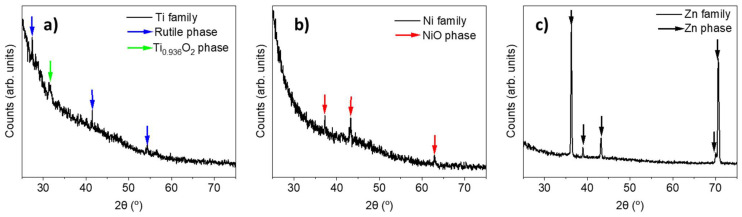
(**a**) XRD spectrum of the Ti family; peaks are marked with arrows of different colours: blue for rutile and green for a non-stoichiometric phase; (**b**) XRD spectrum of the Ni family; (**c**) XRD spectrum of the Zn family.

XRD spectra of the samples were performed at grazing angle. However, due to the cylindrical geometry of the samples and the surface of the layers, the spectra may seem noisy. Figure 6a shows the spectrum of the Ti samples. In this case, we discovered peaks from two phases: a rutile phase (blue) and a non-stoichiometric Ti_0.936_O_2_ phase (green). The more intense peaks (mainly 27.37° and 41.42°) are those which belong to the rutile phase (Figure 5b), from which we can estimate its lattice parameters, a = 4.609 Å and c = 2.934 Å, in consonance with [25]. Figure 6b displays the XRD spectrum of the Ni samples. There, the peaks marked with a red arrow (37.22°, 43.26°, and 62.91°) can be ascribed to the cubic NiO phase (whose structure is shown in Figure 5c). The calculation of lattice parameters for cubic NiO from XRD data yields a = 4.183 Å, which agrees with the bibliography [26]. In the case of the Zn/ZnO system, the oxide layer is thin (≤10 µm) and the XRD spectrum shows only the peaks characteristic of Zn (Figure 6c); nevertheless, the presence of the oxide layer was assessed using X-ray microanalysis, as previously discussed.

**Figure 7:**
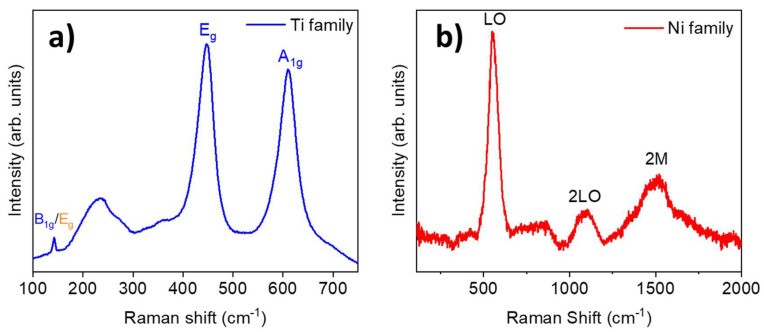
Raman spectra of (**a**) the Ti family. Rutile modes are written in blue, while anatase modes are written in orange; (**b**) the Ni family.

We also assessed the μ-Raman spectra of samples from the Ti and Ni families; Figure 7 shows typical spectra for each family. Spectra were taken from the central region of the oxide layer of the wires, since a better oxidation of the layers is expected in this zone. The Raman spectra of the Zn family (not shown) were too noisy, and no information could be extracted from them. It should be borne in mind that the ZnO layer was very thin, and the depth penetration of the beam was likely larger than the thickness of the ZnO layer itself. In these conditions, the energy would be mainly deposited in the metal core, from which no Raman signal is expected. The main peaks of the Raman spectrum of the Ti family are located at 144, 234, 447, and 612 cm^−1^. The peak at 144 cm^−1^ is attributed to both the rutile phase (B_1g_ mode) and anatase (E_g_ mode) [27,28,29]. Peaks around 447 and 612 cm^−1^ correspond to the E_g_ and A_1g_ rutile modes. The origin of the band at 234 cm^−1^, with a shoulder on 274 cm^−1^, is not clear yet. It is usually ascribed to higher-order Raman modes [27,28,30] or to some kind of disorder [29]. The Raman spectra, along with the fact that the most intense peaks from XRD spectrum were from rutile, allow us to conclude that the TiO_2_ phase is almost entirely rutile. In the case of NiO, the main peaks are approximately located at 553, 1092, and 1509 cm^−1^. The first of them is associated with an LO mode in cubic NiO and the second is related to a 2TO mode [31,32]. The wide band around 1500 cm^−1^ is due to two-magnon (2M) excitation [31,32]. The presence of these Raman modes confirms that the cubic phase of NiO is grown, as suggested by XRD patterns.

**Figure 8:**
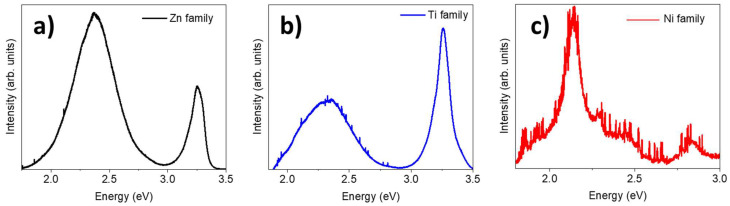
PL spectra from (**a**) the Zn family, (**b**) the Ti family, and (**c**) the Ni family.

Figure 8 shows typical µ-photoluminescence spectra (µ-PL) of the Zn, Ti, and Ni families. Figure 8a shows a typical spectrum of ZnO with two clearly resolved bands: one centred at 3.2–3.3 eV, associated with the near-band edge emissions of ZnO, and a wide band (2–3 eV) in the visible region. This band is very complex because it exhibits shoulders at 2.1, 2.25, 2.3. 2.55, and 2.84 eV and is commonly associated with lattice defects: the most important of them are vacancies and interstitials of oxygen and zinc [33]. The presence of defects related to oxygen vacancies and interstitials, similar to vacancies and interstitials of metallic atoms, are common to metal oxides, meaning similar visible bands can be expected in other metal oxides [34,35,36,37,38]. The band attributed to the near-band gap is asymmetric and seems to be left-shifted from the centre, which could be due to the presence of superficial states whose energy levels are a few meV below the bandgap. Finally, the relative intensity of the visible band is higher than the intensity of the near-band edge. This fact suggests two hypotheses. Either the ZnO layer is not thick enough to be emissive, as the cross-section EDX maps show, or the density of defects is high enough to overcome the ZnO near-band edge in intensity. In Figure 8b we observe the spectrum of Ti samples, with the UV peak corresponding to the TiO_2_ near-band edge [39] and a wide band in the visible region ascribed to trapped carriers in midgap states (usually created by defects in the lattice) which recombine themselves with free carriers [39,40]. In this case, the higher intensity of the TiO_2_ near-band edge can be attributed to the larger thickness of the oxide layer. The more TiO_2_ is grown, the more the near-band edge transition is favoured. Moreover, the visible band is similar to that of ZnO and is associated with oxygen and metallic vacancies and interstitials, as previously mentioned. In this case, the shoulders were visually located at 2.1, 2.15, 2.25, and 2.35 eV. Finally, Figure 8c shows a PL spectrum of the Ni family. This spectrum is noisier than the others because nickel oxide is known to have poorer luminescent properties. Here, we can see two main bands centred at 2.15 and 2.85 eV and a continuum of intensity around 2.3–2.5 eV. Previous studies have determined that nickel vacancies play a more important role than other defects, such as oxygen vacancies [41,42]. This hypothesis is plausible in our case, since the band corresponding to 2.15 eV, the most intense in the spectrum, is usually attributed to nickel vacancies, whereas the second band centred at 2.8 eV can be ascribed to oxygen vacancies [41]. The continuum emission at 2.35–2.37 eV is often reported in the bibliography as a green band also generated by nickel vacancies [43]. In our case, this band may have been redshifted to 2.15 eV due to the presence of native defects or the particle size distribution [44]. The reason why the near-band edge does not appear in Figure 8c could be the particle size distribution, which could cause a shift of the near-band edge (usually 3.47–3.72 eV [43]) towards larger energies (4.3 eV), as reported by [45].

**Figure 9:**
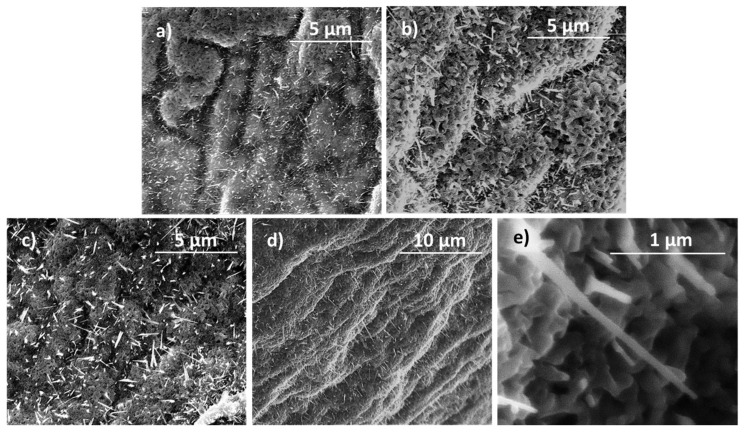
SEM images of the surfaces of (**a**) Zn1, (**b**) Zn2, (**c**) Zn3, and (**d**) Zn4-E, and (**e**) detail of Zn3.

In addition to the formation of the oxide shell, the growth of micro- and nanostructures on it has been also investigated in all the families. Figure 9a,b show images from Zn1 and Zn2 topography. Both surfaces are covered by crystalline randomly oriented needles, as reported in [4,46], and those of Zn2 are bigger those of Zn1. From that, we can conclude that, at a fixed current, time impacts the length of the needles. Figure 9c,d show images of Zn3 and Zn4-E to compare the effect of the external electrical field on the needles grown. Some nanostructure needles are shown in Figure 9e. A brief sampling exhibits that the average length of these microstructures is approximately 1.5 μm. While needles in Zn3 are randomly oriented, those in Zn4-E mainly point in a fixed direction (down right). The main reason for the alignment of these needles is the establishment of a preferential minimum-energy direction imposed by the electric field. The settlement of the electric field is a constant source of infinite energy during the growth, so this direction minimizes the energetic cost for needles to grow. In other words, the nucleation of needles in this direction occurs in this way because the system gets “free energy” from the electric field and growth in any other direction would cost more energy. The number of needles does not seem to change with the application of the field.

**Figure 10:**
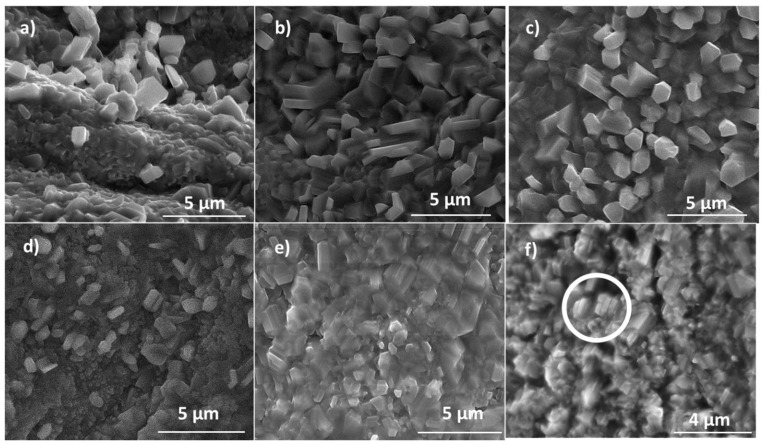
SEM images of the surfaces of (**a**) Ti1, (**b**) Ti2, (**c**) Ti3, (**d**) Ti4, (**e**) Ti5, and (**f**) Ti6-E. The white circle in (**f**) highlights the coalescence of blocks by eliminating boundaries.

Figure 10a–c show details of the Ti1, Ti2, and Ti3 surfaces. As shown, there are some randomly oriented prism-like structures whose size does not appear to change much. However, the number of structures increases with time. The same fact applies to Figure 10d,e due to the boost of needles on the surface of Ti5 in relation to Ti4 under the same current conditions. Nevertheless, it is worthwhile to compare Figure 10c,d to samples Ti3 and Ti4. Apart from the smaller number of crystalline structures grown on Ti3, they seem to be bigger, better formed, and more clearly defined than those grown on Ti4. Since Ti3 was grown at higher current but in less time than Ti4, we can conclude that, in this case, current conditions are more critical than time. Finally, Figure 10e,f show the effect of an external applied field on samples Ti5 and Ti6-E, respectively. No differences between the microstructures are observed, but those of Ti6-E appear in a manner that suggests a favoured growth axis. The circle inset in Figure 10f shows that prisms tend to coalesce. This fact might be imputed to the application of the external field: the polar faces can be merged to minimize the total energy of the system, as will be shown in the scheme of Figure 12b.

**Figure 11:**
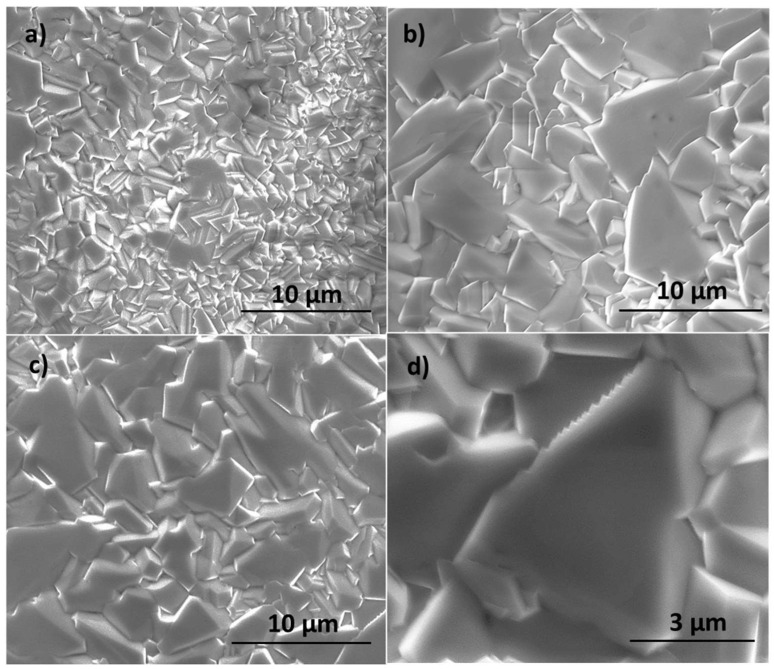
SEM images of (**a**) Ni1, (**b**) Ni2, and (**c**) Ni3-E; (**d**) detail of the saw-toothed edge of a Ni3-E grain.

**Figure 12:**
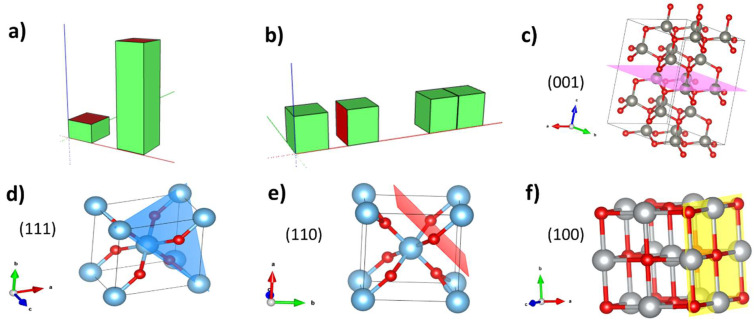
Mechanisms to minimize the total energy of the crystal: (**a**) by growing perpendicular to more surface-energetic faces (red faces), so that the total energy of the crystal is diminished by growing less surface-energetic side faces (green faces); (**b**) by joining two different crystals on the most surface-energetic faces (red faces); (**c**) (001) plane on wurtzite ZnO; (**d**) (111) plane on rutile, (**e**) (110) plane on rutile, and (**f**) (100) plane on cubic NiO. The arrows in (**c**–**f**) show the coordinated axis of each cell.

Figure 11a shows the morphology of Ni1, while Figure 12b,c, show those of Ni2 and Ni3-E. In this case, unlike in the Zn and Ti families, no elongated microstructures grow on the NiO layer, although the formation of microcrystals is observed, resembling the morphologies expected for samples grown using conventional thermal treatments [23]. This fact could be imputed to the cubic lattice of NiO. As previously mentioned, the existence of several equivalent preferential growth directions would explain why the growth is similar in all directions and, in particular, why there are no height differences between them. Time also seems to determine the size of grains: those from Ni2 samples (Figure 11b) are bigger than those from Ni1 (Figure 11c). To explain this fact, we suggest that small grains coalesce as time passes, which would be in concordance with typical sintering processes. The application of an electrical field appears to have little effect on the morphology, as predicted, since Ni2 and Ni3-E (Figure 11c,d) show no significant differences between them. However, it could influence the boundaries between grains. Figure 11d shows a detail of a triangular grain. Unlike grains of Ni1 and Ni2, the upper grain boundary of this one is saw-toothed. Moreover, the distance between spikes is nearly always the same. This geometry has not been observed in other grains of Ni samples but only in Ni3-E. The main hypothesis that we sustain is that this spiky boundary is grown in this way to minimize the surface energy of the boundary due to the existence of an energetically favoured direction created by the electric field.

It is a well-established fact that the morphology of the structures is determined by the existence of privileged growth directions. The preferential directions are those that minimize the area of the most energetic faces so that the surface energy is minimized. For instance, in ZnO, the basal planes have a higher energy; therefore, to minimize the surface area, the growth occurs along the c-axis. In contrast, the presence of several energetically equivalent faces would imply the lack of a preferential growth direction. However, an external energy supply could modify the growth conditions. In crystals with polar planes, i.e., planes in which only one type of ion is exposed, the presence of an external field could favour certain growth directions to a greater extent. Figure 12a depicts a basic scheme of this situation. Another mechanism involving the polarity of the faces could be the coalescence of different crystals at their polar most surface-energetic faces to minimize the energy of the system, as shown in Figure 12b. In ZnO, the preferential growth direction is perpendicular to the basal (001) faces (Figure 12c), which are polar [47]. In titanium oxide, the preferential growth directions are (110), (111), and (311), although the most stable is (110) [48]. Figure 12d,e shows a detail of the (110) and (111) planes on a rutile cell. As can be seen, both faces are polar because they only contain atoms of the same element, and, consequently, the net charge of the plane cannot be zero. From that we may conclude that the electric field would induce additional effects on the morphology. The preferential growth directions for NiO are (100) and (110) [49]. The (100) plane, shown in Figure 12f, is non-polar, as it contains nickel and oxygen atoms, and it has some equivalent planes such as (010) and (001); consequently, it is not expected that these growth directions are favoured by an external field. This simple model can explain why Zn needles are aligned by the action of the field, Ti blocks coalesce in presence of it, and Ni grains are not substantially changed by its application.

## 4. Conclusions

Zn/ZnO, Ti/TiO_2_, and Ni/NiO core–shell structures were grown via the thermal oxidation of pure metal wires. The thickness of the layer was calculated by averaging the radii measured at different points of the shell. For Zn/ZnO wires, the thickness was 10 μm; for Ti/TiO_2_ wires, it was about 30 μm; and for Ni/NiO, it was roughly between 40 and 50 μm. In all composites, we obtained an oxide shell supported by the presence of the K_α_ O line in EDX spectra. The other peaks which appeared are characteristic of each material. The crystalline phase of TiO_2_ composites is mainly rutile, while NiO one is cubic, evidenced by the XRD and Raman spectra. For ZnO, the phase could not be determined via structural analysis; however, PL investigations suggested the existence of crystalline areas within the oxidized layer. Luminescence spectra of ZnO and TiO_2_ show a broad visible band, typically associated with lattice defects, and another UV band related to the near-band edge, while in NiO the bands could be mainly related to nickel vacancies. The morphologies of the Zn, Ti, and Ni families were studied, and critical factors such as current, time, and the application of an external electric field were investigated to optimize the growth conditions. The Zn family forms needle-like structures, the Ti family creates prism-like structures, and the Ni family does not generate structures upwards, but the surface shows microcrystals similar to those normally synthesized in traditional sintering treatments. Along general lines, we can claim that current has more of an influence than time on crystalline quality, although small variations are also observed when time is modified in identical current conditions. The external electric field has an important role in changing the morphologies of ZnO and TiO_2_ composites because of the establishment of a preferential growth direction. In the Zn family, needles are aligned with the field because it favours a minimal-energy growth direction; in TiO_2_, the application of an external field could promote the coalescence of blocks at polar preferential-growth faces. In the Ni family, the external field does not affect the morphology of grains but it influences the formation of spiked edges.

## Figures and Tables

**Table 1:** Detailed conditions of growth for all samples sorted according to each family, where we specify the intensity, circulation time of the current, and whether an external electric field was applied during the growth.

**Zn family**	Intensity	Time	Electric field on/off
Zn1	3.8 A	35 s	Off
Zn2	3.8 A	60 s	Off
Zn3	3.8 A	80 s	Off
Zn4-E	3.8 A	80 s	On
**Ti family**	Intensity	Time	Electric field on/off
Ti1	2.7 A	30 s	Off
Ti2	2.7 A	60 s	Off
Ti3	2.7 A	100 s	Off
Ti4	2.5 A	180 s	Off
Ti5	2.5 A	300 s	Off
Ti6-E	2.5 A	300 s	On
**Ni family**	Intensity	Time	Electric field on/off
Ni1	5.5 A	30 s	Off
Ni2	5.5 A	60 s	Off
Ni3-E	5.5 A	60 s	On

## Data Availability

Data are contained within the article.
